# LAI estimation based on physical model combining airborne LiDAR waveform and Sentinel-2 imagery

**DOI:** 10.3389/fpls.2023.1237988

**Published:** 2023-09-29

**Authors:** Zixi Shi, Shuo Shi, Wei Gong, Lu Xu, Binhui Wang, Jia Sun, Bowen Chen, Qian Xu

**Affiliations:** ^1^ State Key Laboratory of Information Engineering in Surveying, Mapping and Remote Sensing, Wuhan University, Wuhan, Hubei, China; ^2^ State Key Laboratory of Geo-Information Engineering, Xi’an, Shaanxi, China; ^3^ Perception and Effectiveness Assessment for Carbon-Neutrality Efforts, Engineering Research Center of Ministry of Education, Wuhan, Hubei, China; ^4^ Wuhan Institute of Quantum Technology, Wuhan, Hubei, China; ^5^ School of Electrical and Electronic Engineering, Hubei University of Technology, Wuhan, Hubei, China; ^6^ School of Geography and Information Engineering, China University of Geosciences, Wuhan, Hubei, China; ^7^ Chinese Antarctic Centre of Surveying and Mapping, Wuhan University, Wuhan, Hubei, China

**Keywords:** Leaf Area Index (LAI), remote sensing, full-waveform LiDAR, physical model, forest canopy, GORT model, data fusion

## Abstract

Leaf area index (LAI) is an important biophysical parameter of vegetation and serves as a significant indicator for assessing forest ecosystems. Multi-source remote sensing data enables large-scale and dynamic surface observations, providing effective data for quantifying various indices in forest and evaluating ecosystem changes. However, employing single-source remote sensing spectral or LiDAR waveform data poses limitations for LAI inversion, making the integration of multi-source remote sensing data a trend. Currently, the fusion of active and passive remote sensing data for LAI inversion primarily relies on empirical models, which are mainly constructed based on field measurements and do not provide a good explanation of the fusion mechanism. In this study, we aimed to estimate LAI based on physical model using both spectral imagery and LiDAR waveform, exploring whether data fusion improved the accuracy of LAI inversion. Specifically, based on the physical model geometric-optical and radiative transfer (GORT), a fusion strategy was designed for LAI inversion. To ensure inversion accuracy, we enhanced the data processing by introducing a constraint-based EM waveform decomposition method. Considering the spatial heterogeneity of canopy/ground reflectivity ratio in regional forests, calculation strategy was proposed to improve this parameter in inversion model. The results showed that the constraint-based EM waveform decomposition method improved the decomposition accuracy with an average 12% reduction in RMSE, yielding more accurate waveform energy parameters. The proposed calculation strategy for the canopy/ground reflectivity ratio, considering dynamic variation of parameter, effectively enhanced previous research that relied on a fixed value, thereby improving the inversion accuracy that increasing on the correlation by 5% to 10% and on R^2^ by 62.5% to 132.1%. Based on the inversion strategy we proposed, data fusion could effectively be used for LAI inversion. The inversion accuracy achieved using both spectral and LiDAR data (correlation=0.81, R^2 = ^0.65, RMSE=1.01) surpassed that of using spectral data or LiDAR alone. This study provides a new inversion strategy for large-scale and high-precision LAI inversion, supporting the field of LAI research.

## 1 Introduction

Leaf area index (LAI) is one of the prime determinants of photosynthesis, which makes it an important quantity controlling physical and biological processes of plant canopies and assessing forest growth potential (Chen and Black, 1992; Barclay and Goodman, 2000). As a fundamental attribute of global vegetation, LAI has been listed as an essential climate variable by the global climate change research community (Fang et al., 2019). The ability to accurately and rapidly acquire LAI is an indispensable component of process-based ecological research facilitating the understanding of gas-vegetation exchange phenomenon at an array of spatial scales from the leaf to the landscape (Zheng and Moskal, 2009).

Remote sensing data provides large-scale, systematic land surface observations consistently over the globe (Pan et al., 2008). With remote sensing technology, LAI can be mainly derived from a variety of sensors including passive optical sensors, active light detection and ranging (LiDAR) instrument, and microwave sensors (Fang et al., 2019). In passive optical sensors, multispectral and hyperspectral sensors provide spectral measurements across the electromagnetic spectrum, which are sensitive to subtle variations in reflected energy and, therefore, have a giant potential for detecting differences in vegetation (Mananze et al., 2018). However, LAI retrieval using multispectral and hyperspectral data has potential problems, such as the low signal-to-noise ratios (SNRs) of some remote sensing data, the “curse of dimensionality” and problems of saturation (Liu et al., 2016). In addition, optical remote sensing is only capable of capturing information from the horizontal canopy, resulting in a lack of information pertaining to vertical canopy (Xu et al., 2022). In active LiDAR instrument, full-waveform LiDAR systems can digitize the entire reflected energy, resulting in complete waveforms from the top of the canopy to the ground which reflect vertical profiles (Lefsky et al., 1999; Mallet and Bretar, 2009). It has been used to estimate LAI based on canopy structure and radiation transfer principles, especially by means of the correlation with the gap fraction (Wang and Fang, 2020). The primary advantage of LAI estimation using full-waveform LiDAR lies in its ability to capture detailed structural information beneath the canopy through the complete energy waveform, thereby mitigating estimation errors stemming from leaf aggregation. However, the high-density data obtained from airborne LiDAR is limited to the measurement range, whereas spaceborne full-waveform LiDAR data is characterized by its substantial volume. Multi-source remote sensing data have their own advantages and disadvantages in LAI estimation. On this basis, researches on using multi-source remote sensing data fusion to estimate LAI is becoming a research hotspot for which it can give full play to the advantages of different remote sensing data (Clevers and vanLeeuwen, 1996; Yang et al., 2011; Ma et al., 2014; Qu et al., 2015).

Existing methods for fusing spectral and LiDAR data are mostly based on empirical models. The empirical model directly relates inputs to outputs by pure statistical means, the advantage of which lies in its simplicity (Weiss et al., 2020). Thomas et al. (2011) estimated LAI with LiDAR and multispectral data by constructing fused LiDAR-optical indices. Ma et al. (2014) combined LiDAR data with the MODIS and MISR products to retrieve canopy height and LAI by multivariate linear regression model and geometric-optical mutual-shadowing (GOMS) model. Luo et al. (2019) estimated maize LAI using the combined hyperspectral imagery and LiDAR pseudo-waveforms by random forest (RF) regression algorithm. Zhang et al. (2022) estimated the LAI of a short-crop using UAS-based SfM and LiDAR point clouds, as well as the spectral information from multispectral imagery. Zhang et al. (2023) used six typical machine learning algorithms to construct prediction models of LAI, among which the XGBoost model showed the best performance. It also showed that the fusion of data could significantly improve the predictive ability of the models. However, the empirical model is strongly grounded with a large amount of statistical data and can only be applied within a relatively localized area because their performance is highly dependent on vegetation types, canopy structures, sensors, and temporal change (Xu et al., 2020). Empirical models, even superior deep learning models, can also suffer from statistical problems such as overfitting (Verrelst et al., 2015; Neinavaz et al., 2016).

In contrast to empirical methods, the physical model can be better generalized and its physical principles is helpful for the analysis of fusion mechanism (Myneni et al., 1997; Verrelst et al., 2019; Kennedy et al., 2020). Based on LiDAR waveform, commonly used models include gap fraction models (homogeneous canopy or clumping-aware canopy) and three-dimensional (3-D) radiative transfer models. Within multiple gap fraction models, gap fraction can be performed by directly computing its aggregate value (Luo et al., 2013; Fieber et al., 2014; Tseng et al., 2016; Jiang et al., 2022) or by considering the vertical accumulation of gaps within the canopy layers (Ni et al., 1999; Yang et al., 2019). Gap fraction models, with minimal input parameters, streamline LAI computation through forward modeling (Zhao et al., 2011). The geometric-optical and radiative transfer (GORT) model, one of the gap fraction models, capitalizes on the capability of lidar waveform data to characterize the underlying canopy structure (Ni-Meister et al., 2001). Accurate vertical profiles of LAI can be derived by GORT (Tang et al., 2012). This methodology has been employed in the derivation of products for the spaceborne lidar GEDI and exhibits a strong applicability of large-scale LAI estimation (Tang et al., 2014; Wang et al., 2023). Regarding the 3-D radiative transfer models such as Discrete Anisotropic Radiative Transfer (DART), they are proficient in simulating lidar waveforms effectively (Gastellu-Etchegorry et al., 2016; Gastellu-Etchegorry et al., 2017). However, due to its multitude of model parameters and complex scenarios, time-consuming aspects arise during the inversion process. Taking the above reasons into consideration, we consider conducting a data fusion inversion study based on the GORT model. It is still a challenge to combine spectral imagery and LiDAR waveform for LAI retrieval based on physical models. In LAI estimation, spectral features including sensitive bands’ reflectance and spectral indices, play a pivotal role in accuracy (Potithep et al., 2013; Liang et al., 2015). Waveform information such as height, echo energy ratio and leaf coverage are key parameters of LAI estimation using full-waveform LiDAR (Pope and Treitz, 2013; Ma et al., 2015). Improving the physical model to use the above parameters so as to make use of the respective advantages of multi-source data is the key for LAI estimation using both spectral and LiDAR data. In addition, the accuracy of the physical model is susceptible to the initial assignment value of model parameters and the quality of the input data (Houborg et al., 2007). Adjusting the input parameters of the model based on the study area and source data is also an important measure to ensure the accuracy of LAI estimation.

In response to the above problems, the main objectives of this paper are: (1) Developing an estimation strategy based on GORT model to achieve data fusion. (2) Extracting accurate parameters for LAI estimation from both spectral and LiDAR waveform data. (3) Assessing the performance of the joint data fusion for LAI estimation. Specifically, for the first time, we attempt to fuse spectral and waveform data within the GORT model, achieving a joint LAI estimation. We enhance the waveform decomposition method to extract more accurate waveform parameters. Furthermore, on the basis of the existing retrieval, we improve the model input parameter canopy/ground reflectivity ratio ( $\rho_{v}/\rho_{g}$ ) to make it more suitable for the large-scale forest with the heterogeneity of the spectrum. The significance of this study lies in providing novel insights into the fusion of active and passive remote sensing data, thereby contributing to the enhancement of accurate large-scale LAI estimation.

## 2 Study area and data

The study area is located in Harvard Forest, a 4000 acres forest in Petersham, Mass., which is now among the most studied forests in the world. As a critical node in USA national ecological network (LTER, NEON and ForestGEO), Harvard Forest department gathers and produces various datasets from its ecological scientific researches. At the same time, many remote sensing ecosystem projects choose Harvard Forest as study area, which provide multi-source remote sensing data. For these reasons, we chose the Harvard Forest to carry out remote sensing research of multi-source data.

### 2.1 LVIS airborne LiDAR data

NASA’s Land, Vegetation, and Ice Sensor (LVIS), is an airborne, wide-swath, full waveform imaging laser altimeter system, which emits 1064nm wavelength laser pulses to collect data on surface topography and 3-d structure with medium 25m footprint.

During summer 2021, LVIS operated as a NASA Facility to calibrate and validate the space-based LiDAR sensor GEDI (Global Ecosystem Dynamics Investigation) by conducting overflow ground tracks over the Eastern United States and French Guiana. The LVIS Classic instrument was flown on Gulfstream V at a flight altitude of 41,000’, covering Harvard Forest completely on August 6, 2021. The data products of LVIS include Level 1B Geolocated LVIS Waveforms (HDF format) and Level 2 Geolocated Surface Elevation and Height Product (ASCII Text format), from which ecosystem structure parameters can be derived (Blair et al., 1999; Blair and Hofton., 2020).

In this research, we used data products of the LVIS flight on August 6, 2021, obtained from ‘https://nsidc.org/data/LVISC1B/versions/1’. From these, we extracted multiple parameters for each pulse into a comprehensive dataset (.csv). The parameters included: laser shot (shotnumber), longitude (lon), latitude (lat), elevation of the highest detected signal (zt), elevation of the lowest detected mode within the waveform (zg), return waveform (rxwave), signal mean noise level (sigmean).

### 2.2 Sentinel-2 multispectral images

The Copernicus Sentinel-2 mission comprises a constellation of two polar-orbiting satellites. It offers free multi-spectral images with high spatial resolution (four bands at 10 m, six bands at 20 m and three bands at 60 m spatial resolution). The orbital swath width is 290 km with high revisit time (5 days with 2 satellites under cloud-free conditions which results in 2-3 days at mid-latitudes), which support to accurately monitor land surface changes especially vegetation changes and are beneficial to biophysical indicators estimation (Drusch et al., 2012). The Sentinel-2 data has good temporal and spatial resolution with high-quality. Numerous studies have shown that it provides accurate retrieval of LAI. (Korhonen et al., 2017; Hu et al., 2020; Zhou et al., 2020; Sun et al., 2022).

**Figure 1:**
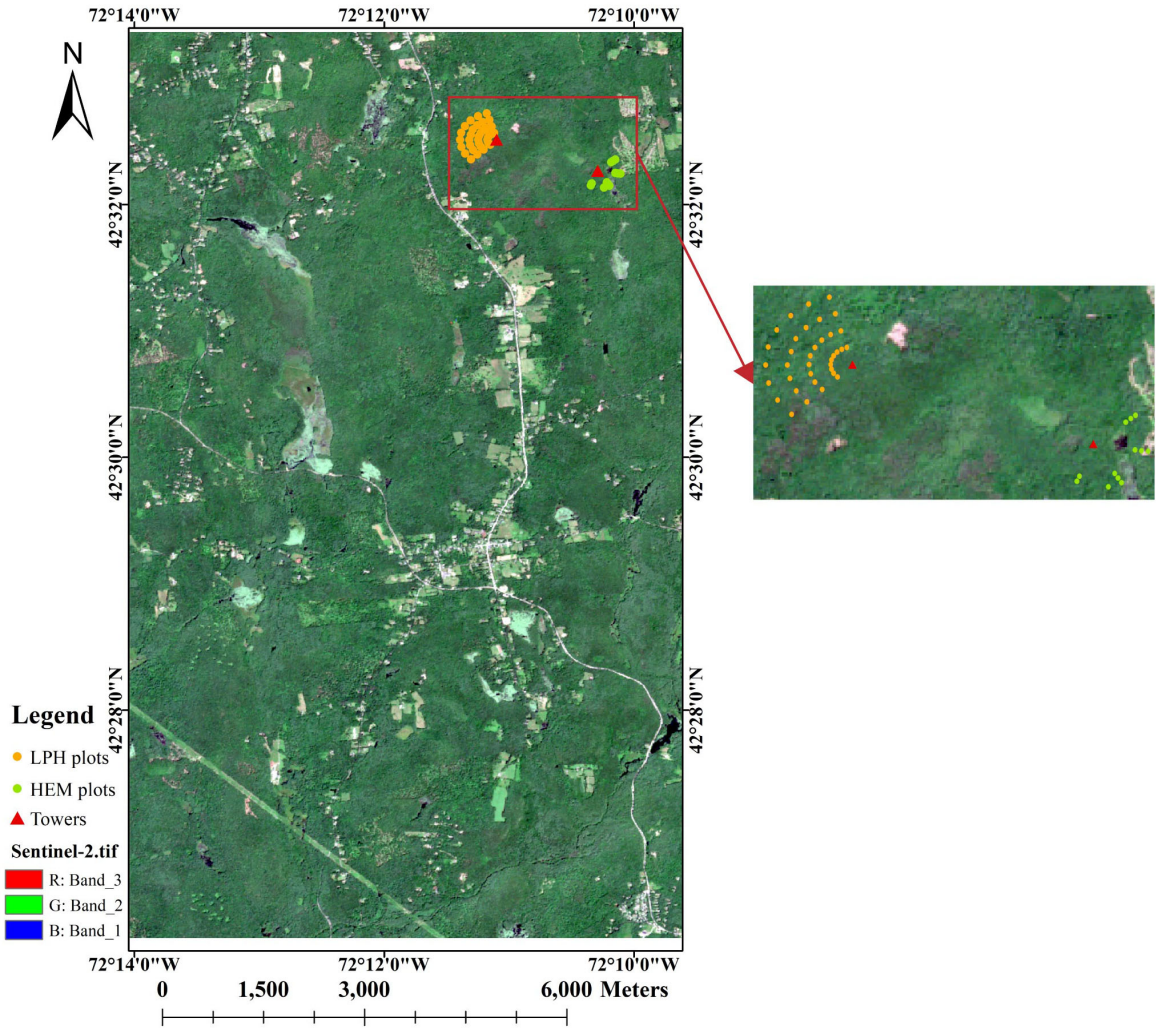
The spatial distribution of ground plots in Harvard Forest (The base map is Sentinel-2 RGB image.).

Sentinel Applications Platform (SNAP), released by European Space Agency (ESA), has been accelerating Earth observation innovation since 2014. It can help to process and analyze Sentinel-2 imagery. In this experiment, the Sentinel-2 image was processed by the Sentinel-2 Toolbox (S2TBX) in SNAP. We acquired an L2A image of Sentinel-2 covering the Harvard Forest on July 31, 2021 (https://doi.org/10.5270/S2_-znk9xsj). The base map of **Figure 1** is the Sentinel-2 RGB image of the study area processed by SNAP. Based on SNAP, we obtained canopy cover map from Sentinel-2 L2A image. Subsequently, using the latitude and longitude information of the laser pulse from the LVIS, we extracted parameters at each pulse point, including spectral reflectance and canopy cover (CC) values.

### 2.3 Ground based LAI in Harvard Forest

The Harvard Forest Data Archive contains various datasets from scientific research at the Harvard Forest, in which HF150 dataset collects Leaf Area Index at Harvard Forest HEM and LPH Towers since 1998 (https://harvardforest.fas.harvard.edu/harvard-forest-data-archive). Leaf area index is measured with the LAI-2000 canopy analyzer with one LAI sensor made at multiple plots within each forest type - usually 12 plots within the old-growth hemlock forest, and 36 plots on Little Prospect Hill (Orwig and Hadley, 2022). The time, distance from tower, compass direction from tower from geographic north, LAI value of each plot is given in the dataset, which help to correlate exact coordinates and values of plots. The spatial distribution of ground plots is shown in **Figure 1**.

## 3 Methods

**Figure 2:**
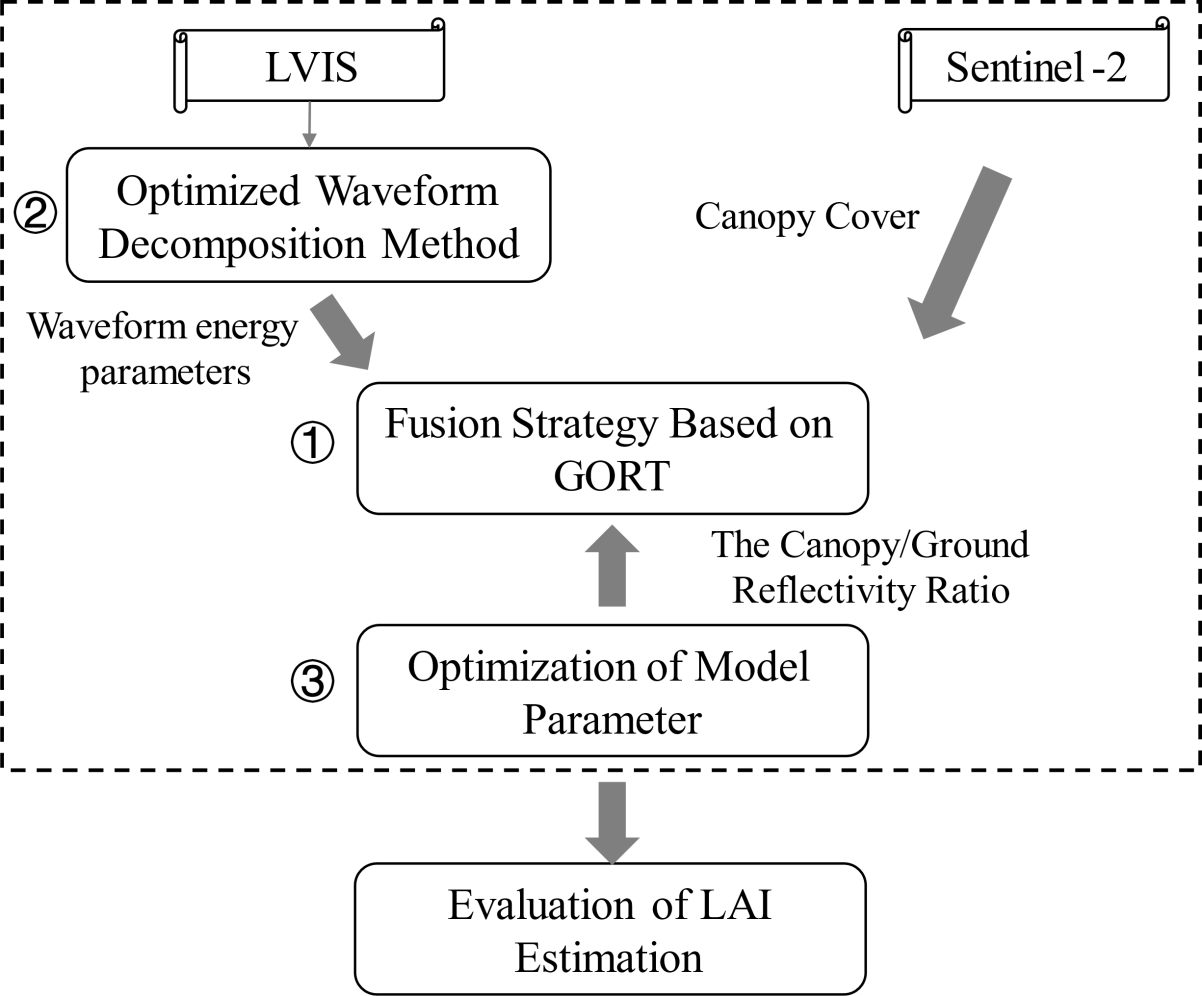
The overview of the methods used in this paper.

To better utilize the advantages of spectral imagery and LiDAR waveform, and considering the scale difference between satellite and airborne data, we first designed a LAI estimation strategy based on a physical model GORT, using airborne LiDAR waveform as the main body and satellite spectral data as support. In order to improve the LAI inversion accuracy, we optimized the model input data and parameters: optimizing waveform decomposition algorithm for more precise waveform energy data, improving the method for obtaining the canopy/ground reflectivity ratio as model parameter to obtain values more consistent with the actual research area. **Figure 2** shows an overview of the methods used in this paper.

### 3.1 A fusion strategy proposal based on GORT model deconstruction

In order to perform joint estimation of LAI using both spaceborne multispectral images and airborne LiDAR waveforms, we decomposed the GORT model and developed a data fusion strategy.

The geometric-optical and radiative transfer (GORT) model is for the bidirectional reflectance of a vegetation cover combines principles of geometric optics and radiative transfer (Li et al., 1995). Ni-Meister et al. (2001) developed a method based on the modified GORT model to derive gap probability and canopy cover from LiDAR waveforms. In the modified GORT model, the cumulative canopy height profile (CHP) was calculated by using a logarithmic transformation of (1-canopy cover). The canopy cover and gap probability could be calculated as follows (Ni-Meister et al., 2001):


(1) $$\begin{array}{c} fcoVer\left(h\right)=1-P_{gap}\left(h\right)=\frac{R_{V}\left(h\right)}{R_{V}\left(0\right)}\frac{1}{1+\frac{\rho_{v}}{\rho_{g}}\;\frac{R_{g}}{R_{V}\left(0\right)}} \end{array}$$

In formula (1), $P_{gap}\left(h\right)$ and fcoVer(h) represented the gap probability and canopy cover percentage above a particular height h within canopy respectively. The terms $R_{V}\left(h\right)$ , $R_{V}\left(0\right)$ and $R_{g}$ were the integrated laser energy returns from the canopy top to height h , from canopy top to canopy bottom, and from the ground return individually. The canopy and ground reflectance were $\rho_{v}$ and $\rho_{g}$ respectively.

**Figure 3:**
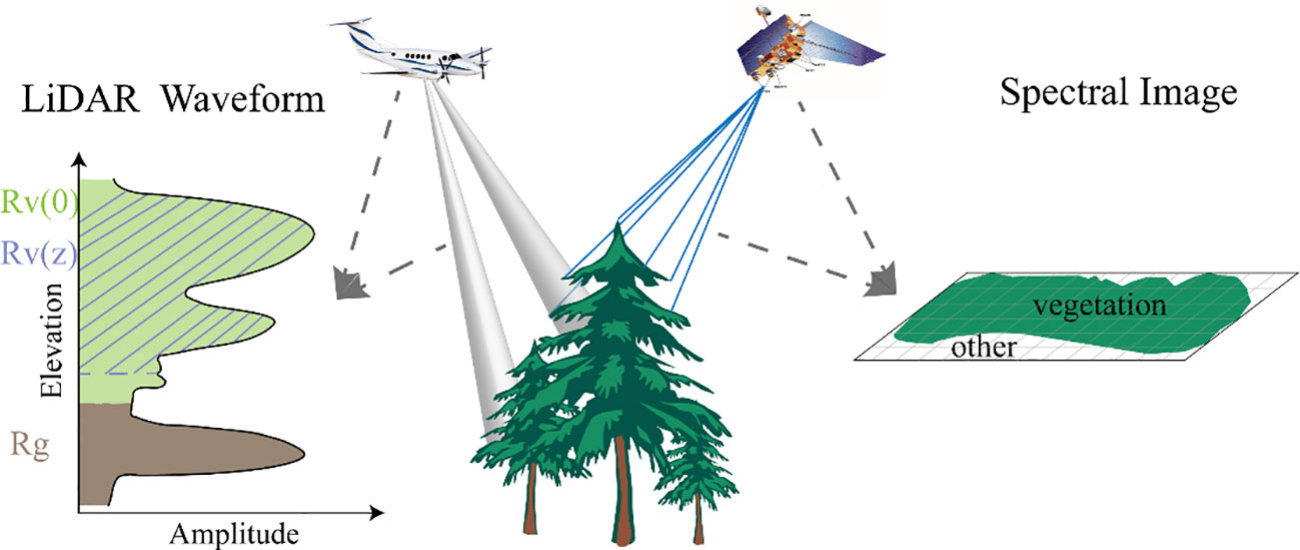
The schematic diagram illustrating the principle of acquiring forest canopy information through active and passive remote sensing methods.

Canopy cover (CC) is typically defined as the extent of ground area covered by the foliage of trees or other vegetation, as projected from a vertical viewpoint onto a horizontal plane (Fiala et al., 2006). From the perspective of remote sensing approaches, two types of canopy cover estimates are commonly derived: metrics describing the 2D horizontal extent of canopy, which is often expressed for a given cover type as a percentage of pixels (Silvan-Cardenas and Wang, 2010); or as 3D LiDAR metrics that represent the transmission of light through the canopy (Morsdorf et al., 2006; Korhonen et al., 2011; Moran et al., 2020). Researches have demonstrated a strong correlation between canopy cover extracted from spectral data and LiDAR-derived inversion values, with the latter frequently exhibiting higher accuracy (Smith et al., 2009; Ma et al., 2017). **Figure 3** shows a schematic diagram of the principles of acquiring forest canopy information using active and passive remote sensing sensors.

Based on the above researches, we assumed that the canopy cover obtained by spectral imagery and LiDAR waveform are equal in this study:


(2) $$\begin{array}{c} fcoVer\left(z\right)=\frac{R_{V}\left(z\right)}{R_{V}\left(0\right)}\frac{1}{1+\frac{\rho_{v}}{\rho_{g}}\;\frac{R_{g}}{R_{V}\left(0\right)}}=FVC \end{array}$$

where fcoVer(z) represented canopy cover percentage above height z . We set the accumulated canopy cover percentage above height z as equal to the estimated canopy cover (FVC) in the pixel at the position of the LiDAR pulse using spectral imagery. z was set to the height corresponding to 80% of the canopy energy in the return. Therefore, the estimate of $\rho_{v}/\rho_{g}$ could be expressed as:


(3) $$\begin{array}{c} \frac{\rho_{v}}{\rho_{g}}=\frac{R_{V}\left(z\right)}{FVC*R_{g}}-\frac{R_{V}\left(0\right)}{R_{g}} \end{array}$$

Based on the above formulas, the canopy/ground reflectivity ratio value of each pulse could be calculated according to LiDAR waveform and spectral imagery.

Tang et al. (2012) deduced the formula of LAI derivation based on the GORT model. The effectiveness of the method has been proved by experiments. Total LAI can be calculated as (Tang et al., 2012):


(4) $$\begin{array}{c} LAI_{total}=\frac{C}{G}\times \ln\left(1+\frac{R_{V}\left(0\right)}{\frac{\rho_{v}}{\rho_{g}}\times R_{g}}\right) \end{array}$$

where C represented the clumping index which adjusted the linear relationship between effective LAI and true LAI (Chen, 1996). We chose the clumping index value of 1.58 for the in Harvard Forest (Tang et al., 2012). G was the projection coefficient and was set to be 0.5 assuming a random foliage distribution within the canopy (Ni-Meister et al., 2001). $R_{V}\left(0\right)$ and $R_{g}$ were the integrated laser energy returns from canopy top to canopy bottom, and from the ground return individually, which were obtained by waveform processing. $\rho_{v}/\rho_{g}$ was calculated as formula (3) using both spectral data and LiDAR waveforms. Based on formula 3 and 4, LAI was estimated by combining spaceborne and airborne data. The strength of this strategy is that we used the physical model to realize the data fusion, and improve the parameter $\rho_{v}/\rho_{g}$ by using the spaceborne spectral reflectance.

### 3.2 Optimization of waveform decomposition method for accurate waveform energy parameters extraction

**Figure 4:**
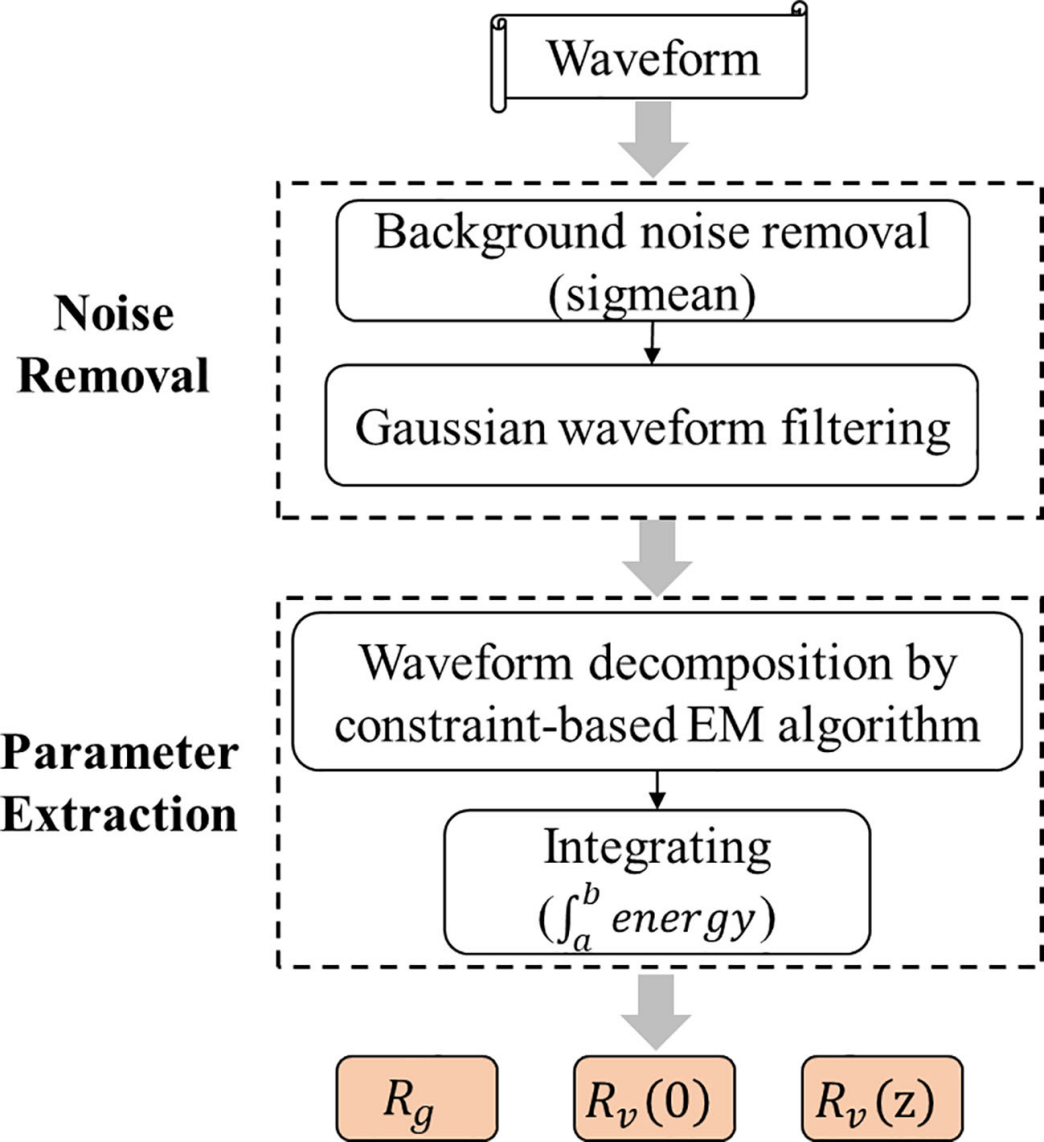
The flow chart of waveform processing ( $R_{v}\left(z\right)$ , $R_{v}\left(0\right)$ and $R_{g}$ are the integrated laser energy returns from the canopy top to height z , from canopy top to canopy bottom, and from the ground return individually.).

Waveform energy parameters are the main input data in LAI inversion based on the GORT model, including canopy backward energy, ground backward energy and waveform energy integral returned at different altitudes, which are useful for segmentation, classification and inversion purposes, in both forested and urban areas (Mallet and Bretar, 2009). Selecting an appropriate processing method to “purify” the original waveform data is vital to extract structure parameters of forest, so as to accurately invert physical parameters of vegetation. In this paper, waveform processing procedure was designed for the processing of the Level 1B Geolocated LVIS Waveforms and Level 2 Geolocated Surface Elevation and Height Product. Specifically, to extract energy parameters more accurately from waveforms, the waveform decomposition algorithm was improved. The processing flow chart is shown in **Figure 4**.

Firstly, the background noise of the echo was removed based on the average noise parameter “sigmean” of each echo calculated in flight provided by LVIS Level 1B product. Values less than the noise threshold was eliminated.

Secondly, the Gaussian filtering algorithm was used to remove other types of noise and smoothed the waveform. The methods of waveform denoising mainly include Gaussian filtering, mean filtering, Fourier low-pass filtering, etc. (Zhang et al., 2020). Gaussian filtering has small time-frequency window area and a simple design, which makes it widely used in the field of signal processing. By measuring the echo denoising effect and adjusting the parameters, a Gaussian filter with better denoising effect was finally selected for echo denoising.

Finally, waveform decomposition method was applied to decompose the echo and extracted effective waveform parameters. Since the backscattered echo signal can be considered as the superposition of multiple Gaussian signals, the Gaussian decomposition method was used to fit the original signal to the superposition of multiple Gaussian function curves (Zhou et al., 2022). The backscattered echo can be expressed as:


(5) $$\begin{array}{c} W\left(t\right)=\varepsilon +\sum_{m=1}^{N_{p}}A_{m}exp\left[-\frac{{\left(t-t_{m}\right)}^{2}}{2\sigma_{m^{2}}}\right] \end{array}$$

In formula (5), W(t) is the amplitude of the waveform at time t ; ε is the bias of the Gaussian waveform; $N_{p}$ is the number of Gaussian components; $A_{m},\;t_{m},\;\sigma_{m}$ are the amplitude, peak position and waveform width of the waveform of the *m*th Gaussian component respectively.

There are two main steps in the waveform decomposition: 1) estimation of the initial parameters; 2) optimization of the parameters and fitting the waveform (Zhou et al., 2021). After extracting accurate initial values of parameters, the commonly used waveform fitting methods include LM (Levenberg-Marquardt optimization algorithm) method (Wagner et al., 2006) and EM (Expectation-Maximization algorithm) method (Persson et al., 2005), the accuracy of which has no significant difference (Zhou et al., 2022). In the global optimization algorithm, waveform components that are too close in distance are prone to being merged during optimization, leading to significant fitting errors. In this paper, in order to address recognition errors caused by some echo components being close to the target, constraints were placed on the values of each peak to improve the accuracy of the fitting based on EM algorithm. The specific approach of the constraint-based EM was designed as follows:

E-step: Compute the posterior probabilities for each component given the data points using Bayes’ rule. The posterior probability for the j th component of the Gaussian mixture model for the  i th data point was given by:


(6) $$\begin{array}{c} \gamma_{ij}=\frac{w_{j}p_{j}\left(x_{i}\right)}{\sum_{k=1}^{K}w_{k}p_{k}\left(x_{k}\right)} \end{array}$$

where $\gamma_{ij}$ represented the probability that the i th data point belonged to the j th component, $p_{j}\left(x_{i}\right)$ was the probability density function of a Gaussian distribution at $\;x_{i}$ of the j th component.

M-step: Update the parameters of the Gaussian mixture model using the posterior probabilities computed in the E-step. The update equations for the means ( $\mu_{j}$ ), and standard deviations ( $\sigma_{j}$ ) of the j th component were given by:


(7) $$\begin{array}{c} P_{\left[a,b\right]}\left(x\right)=\{\begin{array}{cc} a & \;if\;x<\;a\\ x & if\;a\leq x\leq b\\ b & if\;x>b \end{array} \end{array}$$

(8) $$\begin{array}{c} a=\mu_{j_{0}}-\sigma_{j_{0}},\;b=\mu_{j_{0}}+\sigma_{j_{0}} \end{array}$$

(9) $$\begin{array}{c} \mu_{j}=P_{\left[a,b\right]}\left(\frac{\sum_{i=1}^{N}\gamma_{ij}x_{i}}{\sum_{i=1}^{N}\gamma_{ij}}\right) \end{array}$$

(10) $$\begin{array}{c} \sigma_{j}^{2}=\frac{\sum_{i=1}^{N}\gamma_{ij}{\left(x_{i}-\mu_{j}\right)}^{2}}{\sum_{i=1}^{N}\gamma_{ij}} \end{array}$$

where $P_{\left[a,b\right]}\left(x\right)$ was the projection operator that maps x onto the interval [a,b] , $\mu_{j_{0}},\sigma_{j_{0}}$ were the initial values of the mean and standard deviation for the j th component.

Repeat E-step, M-step until convergence was achieved. The constrained EM algorithm for LiDAR waveform decomposition imposed constraints on the parameter range within the optimization problem, leading to enhanced stability and accuracy of the algorithm.

After waveform processing, we identified the last waveform component as the ground component, and the rest as the canopy components. $R_{g}$ was the area enclosed by the amplitude of the last waveform component and the coordinate axis within its start-stop range. $R_{V}\left(0\right)$ was the integral value of the other waveform components within their start-stop range. $R_{V}\left(z\right)$ was the integral value of the waveform from the initial position of canopy component to the height of z .

In this section, we implemented the processing of LiDAR waveforms, especially by adding constraints on the peak positions in the waveform decomposition algorithm to obtain better waveform decomposition results and calculate more accurate waveform energy parameters.

### 3.3 Optimization of model parameter $\rho_{v}/\rho_{g}$ for large scale forest by gridding study area

When using the strategy proposed in Section 3.1 to calculate the LAI of each pulse position, both spectral and LiDAR data are used to calculate the model parameter $\rho_{v}/\rho_{g}$ at this point, that is, $\rho_{v}/\rho_{g}$ varies with the input data in each position. Although the parameter setting strategy is more accurate than taking a fixed value for the entire study area, it causes large data uncertainty and increases the computation of inversion. For example, abnormal waveform of a pulse will result in abnormal calculated value of $\rho_{v}/\rho_{g}$ , thus leading to abnormal LAI inversion results at this point.

To solve this problem, we proposed a method to optimize the model parameter $\rho_{v}/\rho_{g}$ , that was, gridded the study area and calculated the mean value of $\rho_{v}/\rho_{g}$ in each grid. Then the LAI estimation model was constructed by each grid. In a large area, the canopy/ground reflectivity ratio varies with forest environmental conditions. Gridding the study area not only accounts for the spatial heterogeneity within Harvard Forest but also reduces the computational complexity of LAI modeling, thereby mitigating uncertainty arising from anomalous input data. Theoretically, the optimization method is helpful to improve the inversion accuracy.

Specifically, we divided the study area into 2331 rectangular grids (63*37), the coordinate size of each grid was 0.002° * 0.002° (about 36118m^2^). In order to reduce the uncertainty, the statistical method of histogram distribution was used to eliminate extreme abnormal ratio values that without the range of the mean plus or minus three standard deviations in each grid. Then an average reflectivity ratio was assigned for all the pulses in grid to reduce the uncertainty caused by pulse quality. The reflectivity ratio of each grid was the trimmed mean of all pulses in the grid.

## 4 Result and discussion

### 4.1 Waveform energy parameters based on optimized waveform decomposition method

For the purpose of obtaining accurate canopy and ground energy parameters, we processed a total of 187,302 LiDAR waveforms in the study area through denoising, smoothing, and waveform decomposition based on least squares optimization. During this process, the waveform energy parameters Rg and RV obtained using different least squares optimization methods were compared.

The correspondence between the original waveforms and Gaussian model estimates of Rg and  RV using different optimization algorithms is shown in **Table 1**. Overall the correspondence is high for both RV and Rg , the R^2^ of which are all above 0.99. Further comparative analysis between the canopy and ground components reveal that the overall canopy R^2^ is lower than that of the ground. The RMSE for the canopy is significantly higher than that of the ground, with a maximum difference of 35.656. It can be attributed to two main factors. Firstly, the canopy component often has one or more echo components in LiDAR waveforms, whereas the ground component typically only has one. Multiple echo components result in larger errors in waveform decomposition, leading to poorer correspondence of RV . Secondly, due to the significant differences in canopy coverage, the waveform energy fluctuation of canopy is also much larger, resulting in a much higher RMSE compared to the ground.

**Table 1:** Correlation results of waveform and Gaussian model integrals using different optimization algorithms for the ground ( Rg ) and canopy ( Rv ) components of the waveforms.

Waveform energy parameters	Optimization algorithms	R^2^	RMSE
*Rv*	Constraint-based EM	0.991	62.980
	EM	0.990	65.926
*Rg*	Constraint-based EM	0.998	27.324
	EM	0.997	33.766

“Constraint-based EM” means the Expectation-Maximization algorithm with peaks boundary constraints, “EM” means the original Expectation-Maximization algorithm.

Comparing the results obtained from two different optimization methods, it is found that correspondence is higher for both Rg and  RV when the bound of peaks are constrained, compared to the result without parameter constraints (higher R^2^ and lower RMSE). To investigate the reasons for the result, we compare the waveform fitting results of two different optimization methods. The results of waveform decomposition using unconstrained optimization algorithm and optimization algorithm with peak boundary constraints are shown in the **Table 2**. We conducted an average statistical analysis of the fitting results for all waveforms in the study area and found that the constrained optimization algorithm yielded a fitting waveform with $\bar{R^{2}}$ of 0.979, $\bar{MAE}$ of 6.907, $\bar{MSE}$ of 103.016, and $\bar{RMSE}$ of 8.596. The fact that the average $R^{2}$ is almost close to 1 and the small values of the average MAE and RMSE indicate good overall waveform fitting results, suggesting that the model is able to fit the majority of the LiDAR waveforms accurately. The optimization algorithm with no constrain yielded a fitting waveform with $\bar{R^{2}}$ of 0.978, $\bar{MAE}$ of 6.971, $\bar{MSE}$ of 104.740, and $\bar{RMSE}$ of 8.681. Compared with the constrained method, the average MAE, MSE, and RMSE values are higher for the unconstrained method, while the difference of average R^2^ is not significant. This suggests that the overall fitting accuracy of the unconstrained method is lower than that of the constrained method.

**Table 2:** Performance of waveform decomposition using different optimization algorithms.

Optimization algorithms	$\bar{R^{2}}$	$\bar{MAE}$	$\bar{MSE}$	$\bar{RMSE}$
Constraint-based EM	0.979	6.907	103.016	8.596
EM	0.978	6.971	104.740	8.681

“Constraint-based EM” means the Expectation-Maximization algorithm with peaks boundary constraints, “EM” means the original Expectation-Maximization algorithm.

**Figure 5:**
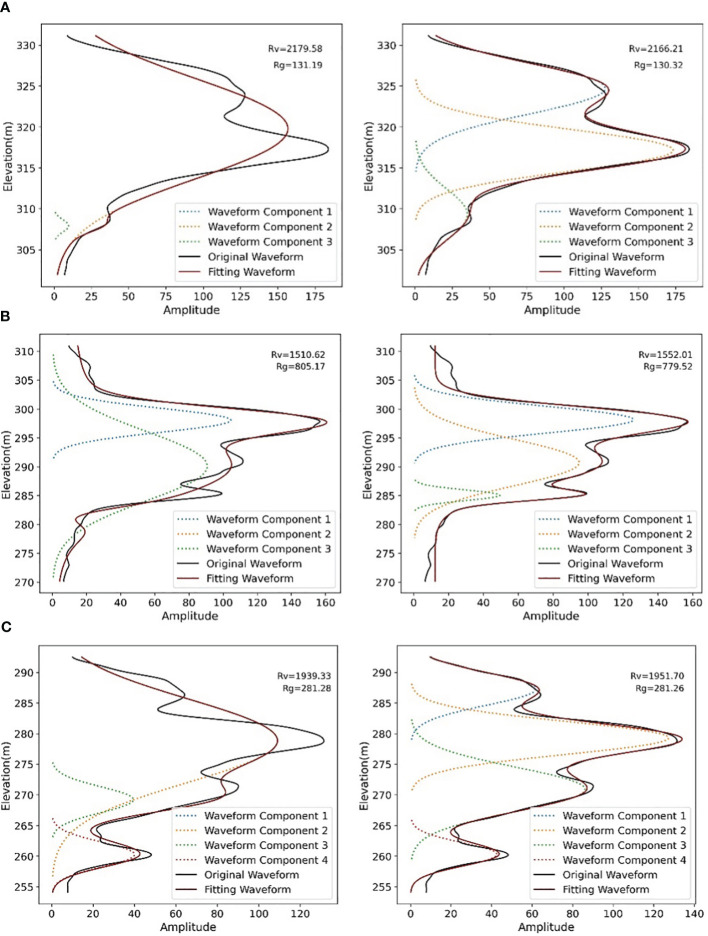
Waveform fitting and waveform energy parameters results using two different optimization methods. **(A)** with two canopy echoes and one ground echo; **(B)** with two canopy echoes and one ground echo; **(C)** with three canopy echoes and one ground echo (The left column: the original Expectation-Maximization algorithm, the right column: the Expectation-Maximization algorithm with peaks boundary constraints).

To further analyze the waveform decomposition result using different methods and its effects on the value of Rg and  RV , we extracted some waveforms with significant differences in inversion accuracy using two different optimization methods (**Figure 5**). The left column shows results obtained by EM method, while the right column shows results obtained by constraint-based EM method. The waveform energy parameters result of the three waveforms are shown in **Table 3**. Waveform (a) contains two canopy echoes and one ground echo. The waveform decomposition using EM method identifies only one canopy echo, and the calculated energy of the canopy echo is 2179.58, which differs significantly from the actual value of 2166.87. The constraint-based EM method identifies two canopy echoes, and the calculated value of 2166.21 is almost the same as the actual value. Waveform (b) contains two canopy echoes and one ground echo, and the second canopy echo and ground echo are combined into one waveform component using the EM method, resulting in a large difference between the canopy and ground energy calculation results of 1510.62 and 805.17 and the actual 1562.44 and 775.86, while the added constraint method does not show this phenomenon. Waveform (c) contains three canopy echoes and one ground echo, which are partially combined by the EM method due to the close distance of each canopy echo, resulting in a large difference between the calculated canopy energy (1939.33) and the actual value (1945.25). The results reveal that the waveform energy parameters calculated by constraint-based EM method are more precise than the unconstrained EM method. The waveform diagram shows that the main reason for the difference in accuracy between the two waveform decomposition methods is that after constraining the peak position of each waveform component, the merge of the closed waveform echoes can be avoided.

**Table 3:** Rg and  Rv of typical waveforms based on different methods.

Typical waveforms	Parameter	Constraint-based EM	EM	Actual value
(a)	*Rv*	2166.21	2179.58	2166.87
	*Rg*	130.32	131.19	128.82
(b)	*Rv*	1552.01	1510.62	1562.44
	*Rg*	779.52	805.17	775.86
(c)	*Rv*	1951.70	1939.33	1945.25
	*Rg*	292.26	291.28	294.82

These results provide evidence that compared to optimization algorithms without parameter boundary constraints, the proposed constraint-based EM method can perform waveform decomposition more accurately and, to a certain extent, avoid decomposition errors caused by waveform components being too close to each other. Based on the waveform decomposition method, more precise RV and Rg can be obtained, providing accurate values for subsequent LAI inversion.

### 4.2 The gridded $\rho_{v}/\rho_{g}$ result

**Figure 6:**
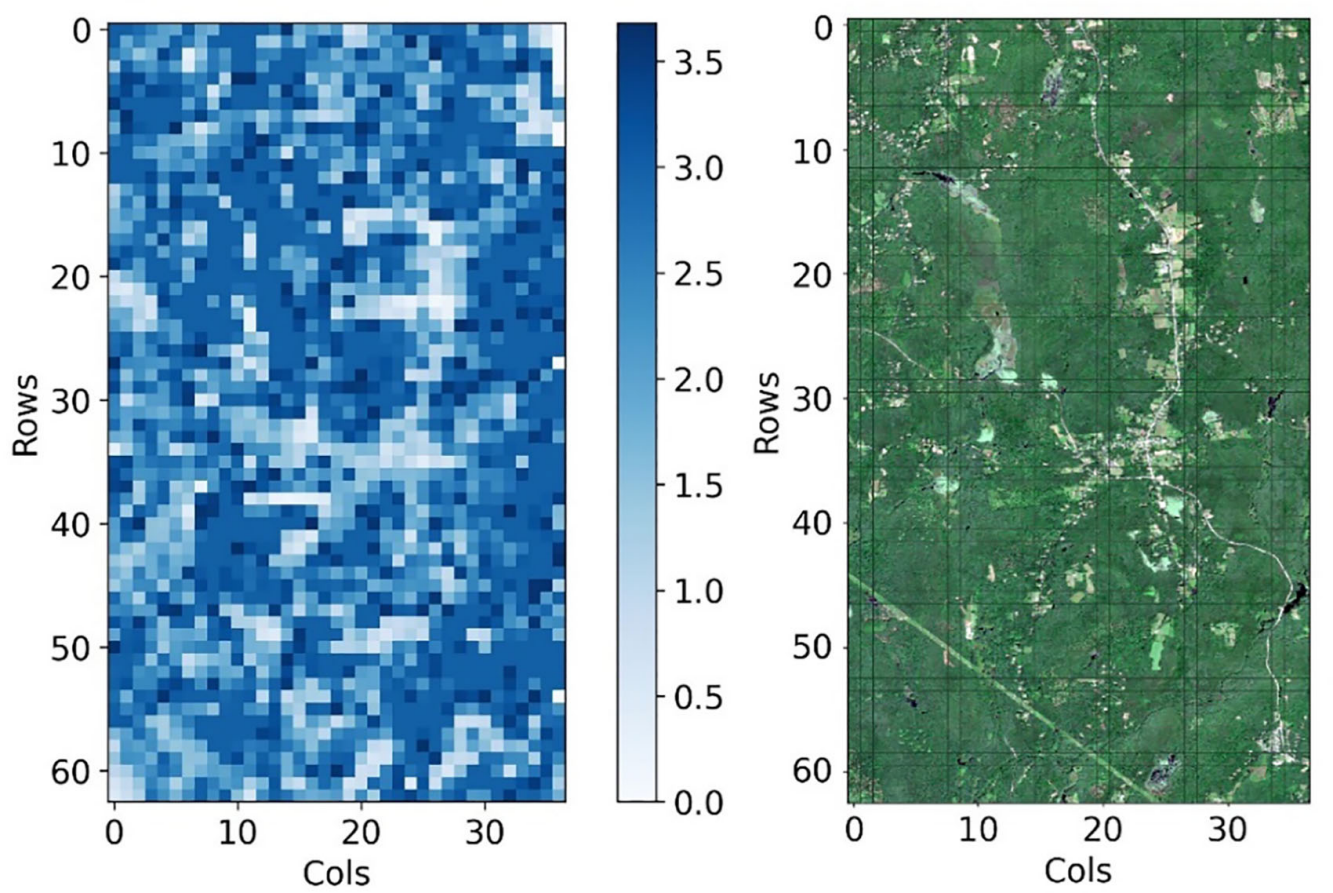
The heat map of gridded canopy/ground reflectivity ratios in the study area (left) (The right image shows the Sentinel-2 RGB image of the study area).

After gridding the study area, the $\rho_{v}/\rho_{g}$ value of each grid is shown in **Figure 6**, and the statistical result is shown in the **Table 4**. Comparing the value heatmap (**Figure 6** left) with the RGB images (**Figure 6** right) of the study area, it can be found that the $\rho_{v}/\rho_{g}$ is correlated with the degree of vegetation coverage. The value of $\rho_{v}/\rho_{g}$ in non-vegetated and sparsely vegetated areas is generally lower than that of areas with higher vegetation coverage. The spatial distribution of $\rho_{v}/\rho_{g}$ coincided with the actual vegetation distribution, indicating the accuracy of the calculation method we proposed. The statistical results show that the final ratio of 2331 grids in the study area is within the range of [0, 3.68], the average value is 2.45, and the root mean square is 0.80.

**Table 4:** The statistics of the canopy/ground reflectivity ratio of each grid.

Parameters	Value
Number of grids	2331
Max	3.68
Min	0.00
Average	2.45
Standard deviation	0.80

In previous studies, Lefsky et al. (1999) suggested using a constant ( $\rho_{v}/\rho_{g}=2$ ) for 1064 nm. Tang et al. (2012) obtained the $\rho_{v}/\rho_{g}$ value of 2.5 at 1064 nm and used it as the mean value for the whole study area. In addition to determining the ratio by empirical field measurements, extracting from LiDAR waveforms using statistical methods (Ni-Meister et al., 2010; Armston et al., 2013) is also a way to calculate this ratio. The value of canopy/ground reflectivity ratio is basically between [0, 3]. Due to the lack of measured data, and no research has used the method of combining spectral and LiDAR data to calculate $\rho_{v}/\rho_{g}$ , it is currently impossible to accurately demonstrate the accuracy of the calculated ratios. However, the average and mean square deviation results show that most of the reflectance ratios are [0, 3] with only a few abnormal values, it can be proved that accurate gridded $\rho_{v}/\rho_{g}$ of the study area can be obtained by this strategy. These provide a new idea for the calculation of $\rho_{v}/\rho_{g}$ .

### 4.3 Comparison of LAI inversion results based on different data and inversion methods

In this section, we explored the LAI retrieval results based on different datasets and different methods for calculating $\rho_{v}/\rho_{g}$ . The accuracy is compared with the existing field measurement data.

**Figure 7:**
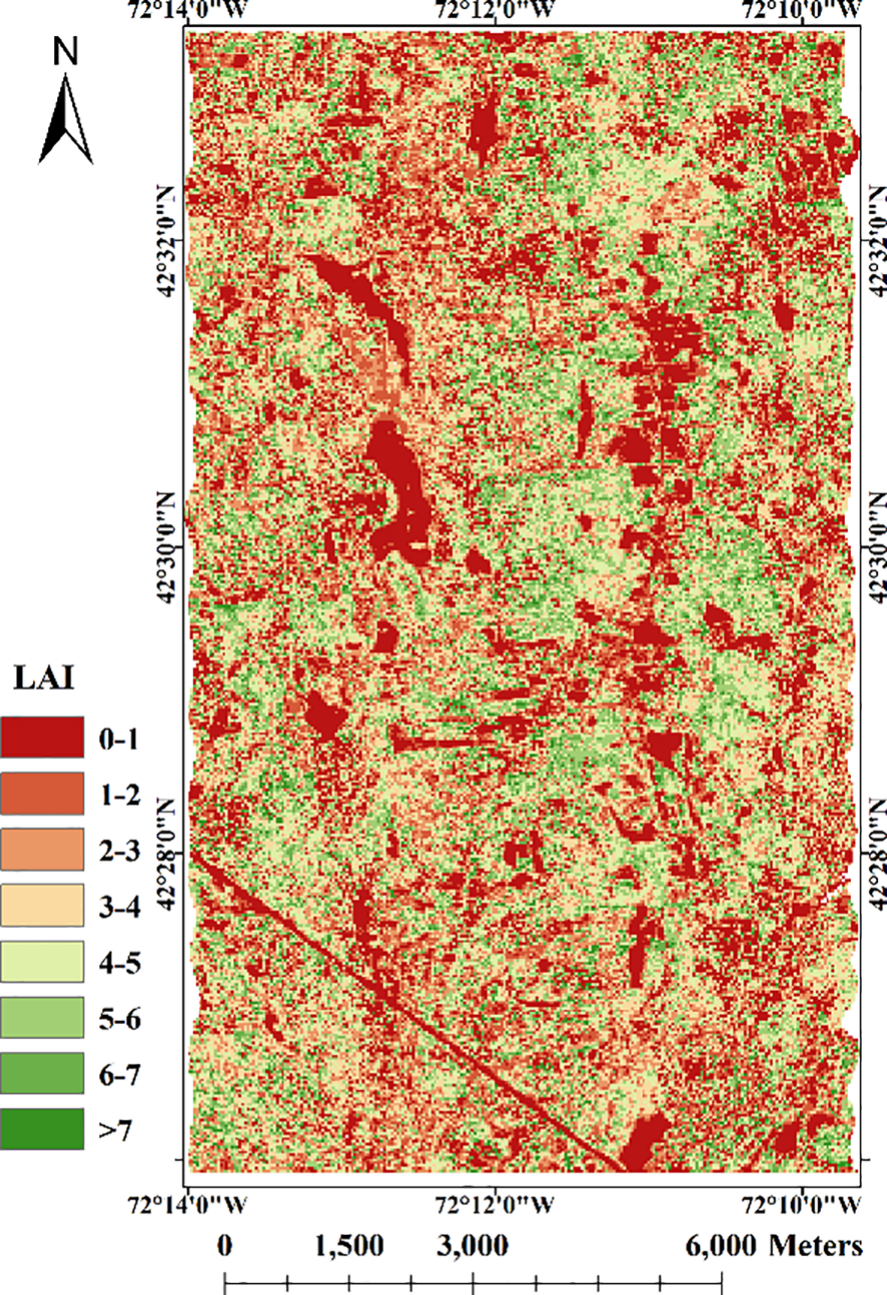
LAI map of the study area using both spectral and LiDAR data.

**Figure 8:**
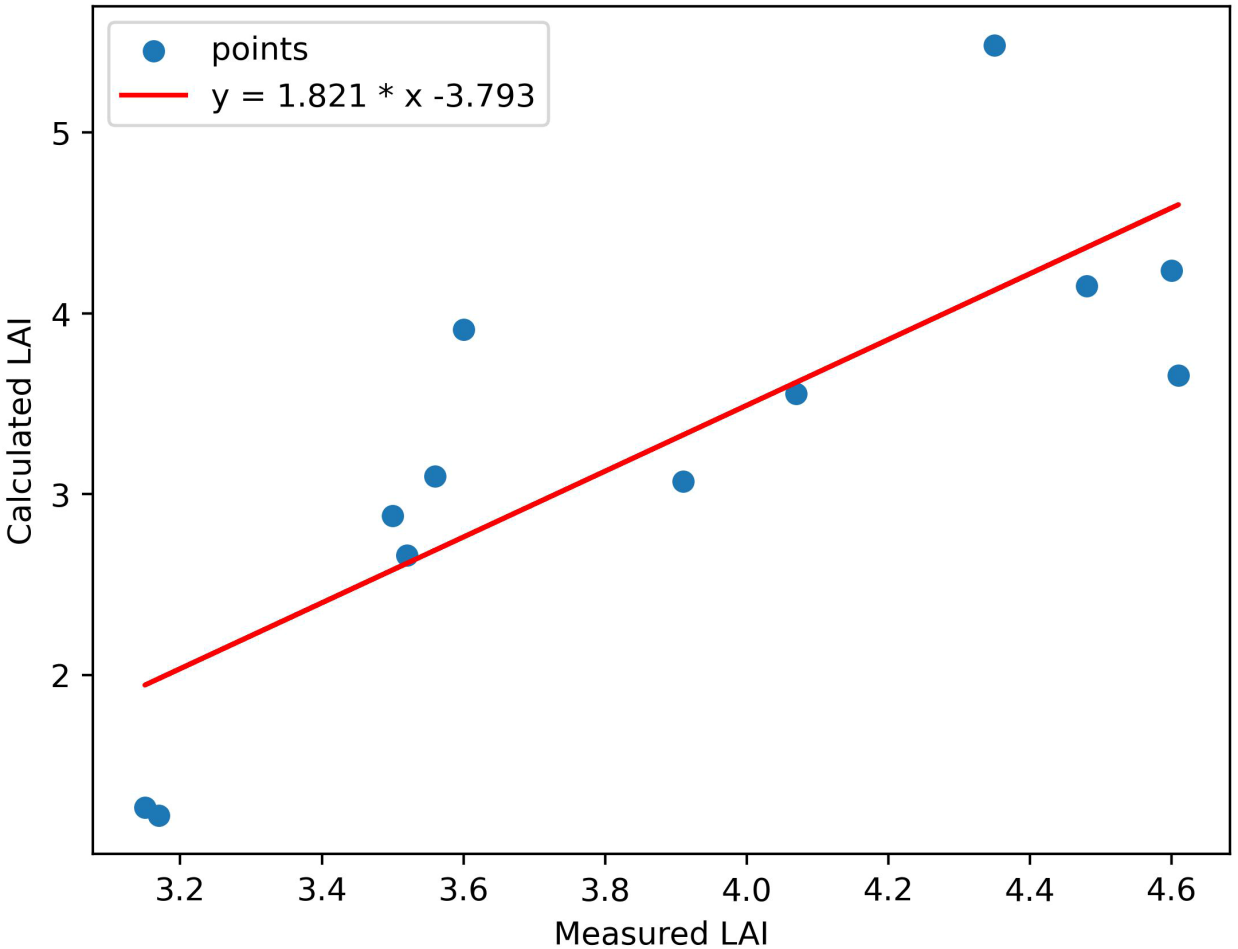
Scatterplots of field-observed LAI against estimated LAI using both spectral and LiDAR data.

Using the fusion strategy we proposed, we obtained the LAI map using LiDAR waveform and multispectral image (**Figure 7**). The result shows that directly using the data of entire study area without land cover classification can estimate LAI well. For example, LAI values of the road in the lower left of this research area are 0. We can clearly distinguish the vegetation and non-vegetation areas from the LAI map. In the vegetated area, the LAI values are generally around 3-7, and reach above 7 in a few dense areas, indicating that the area is relatively heavily forested, which is basically consistent with the actual situation. Comparing the inversion results with the true LAI value provided by Harvard Forest HEM plots, the correlation, R^2^ and RMSE are 0.81, 0.65, 1.01 respectively (**Figure 8**), which shows that the LAI map obtained have high accuracy. The method we proposed to invert LAI by fusing LiDAR and spectral data is feasible.

**Figure 9:**
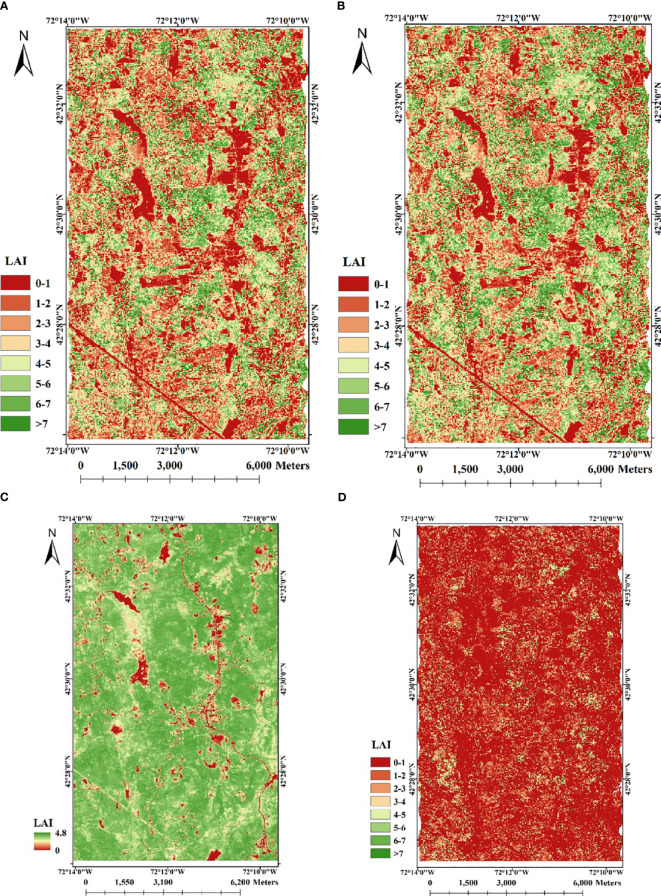
LAI maps of the study area using different strategies. **(A)** by Tang et al. (2012) ’s method; **(B)** by Armston et al. (2013) ’s method; **(C)** by NDVI-LAI relationship model; D: by Yang et al. (2019) ’s method.

We conducted LAI estimation based on four different strategies for comparative analysis. These strategies encompassed two that exclusively utilized LiDAR data, one that solely relied on spectral data, and one that integrated both LiDAR and spectral data. Using only LiDAR data, we reproduced the methods used by Tang et al. (2012) and Armston et al. (2013) respectively. They both performed the inversion based on the GORT model, only some of the parameters in the model were determined in different ways. The LAI estimation maps (**Figures 9A, B**) were performed according to the parameters they set. Using only spectral data, we adopted the most traditional empirical model to construct the linear relationship between NDVI-LAI for LAI inversion using the LAI true value of the Harvard Forest LPH flux tower’s 36 plots (**Figure 9C**). Using LiDAR waveform and spectral data, we employed the method proposed by Yang et al. (2019) and the obtained result was depicted in **Figure 9D**. Based on the true LAI value of Harvard Forest, the accuracy evaluation of the estimation results obtained by various methods was carried out. The overall accuracy results are shown in **Table 5**.

**Table 5:** The accuracy of LAI retrieval results using different methods.

Data	Reference	Model	$\rho_{v}/\rho_{g}$ calculation strategy	$\rho_{v}/\rho_{g}$	Correlation	R^2^	RMSE
LiDAR waveform and Spectral imagery	\	GORT	Fusion strategy (proposed)	Change value	0.81	0.65	1.01
LiDAR waveform	Tang et al. (2012)	GORT	Set experienced value	2.50	0.63	0.40	2.01
LiDAR waveform	Armston et al. (2013)	GORT	Least squares	1.07	0.53	0.28	2.85
LiDAR waveform and Spectral imagery	Yang et al. (2019)	Gap fraction-based model	Set experienced value	2.00	0.51	0.30	2.76
Spectral imagery	\	NDVI-LAI relationship	\	\	0.48	0.25	2.72

\, indicates that the grid value is empty.

**Figures 9A, B** indicates the LAI inversion results using only LiDAR data. Similar to LAI map obtained by the method proposed in this study, the results show obvious differentiation between vegetation and non-vegetation areas. However, the difference lies in that, LAI values of invention are too high in places with dense vegetation only using LiDAR data. By Tang et al. (2012) ‘s method, the LAI inversion result shows the correlation of 0.63, R^2^ of 0.40 and RMSE of 2.01 with ground plot LAI, indicating a moderate correlation between the two. By Armston et al. (2013) ‘s method, the inverted LAI is weakly correlated with the true value, with a correlation coefficient of 0.53, R^2^ of 0.28 and RMSE of 2.85. It shows that the accuracy of the two LiDAR-only inversion methods is lower than that of the fusion of LiDAR and spectral data. The LAI map inverted by the empirical model is shown in **Figure 9C**. The results show that there is a large difference between the LAI inversion results obtained by using only spectral data and those obtained by fusing the two data. Based only on spectral data, the inverted LAI is an underestimate with the highest value being only 4.8. It shows a correlation of 0.48, R^2^ of 0.25 and RMSE of 2.72 with ground plot LAI, which is poor compared to the inversion result that combines the two data. **Figure 9D** illustrates a low accuracy in LAI estimation (the correlation of 0.51, R^2^ of 0.30 and RMSE of 2.76), with LAI values consistently underestimated. This discrepancy may be attributed to the unsuitability of the model for the tree species and data sources in this study area. The original LiDAR waveform used by Yang et al. (2019) is acquired from a large-footprint LiDAR system (70m), which significantly differs from the experimental data used in this study (25m). Comparing various estimation strategies, it is evident that the fusion of both active and passive remote sensing data contributes to improved LAI estimation accuracy. The enhancements we have proposed for the GORT model further enhance LAI estimation precision.

We successfully estimate LAI based on the GORT model combining LiDAR and spectral data with a correlation of 0.81 and R^2^ of 0.65, which shows a large accuracy improvement compared to both LiDAR data alone and spectral data alone. These improvements can be attributed to the addition of spectral data to improve the parameters of the model. Comparing the three inversion methods based on the GORT model, the main difference between them is the $\rho_{v}/\rho_{g}$ value of the model, which may be the main reason for the difference in inversion accuracy. The $\rho_{v}/\rho_{g}$ of the study area is determined as a constant value of 2.5 by experience, which helps to reduce the amount of computation. But this empirical value may not necessarily be applicable to Harvard Forest and a fixed value cannot adequately represent the forest conditions of the entire study area, which may be the prime causes of the low accuracy. The use of least squares methods provides a new approach for the calculation of the ratio, which does not rely on manual measurements, but rather on the energy returned by the LiDAR. Based on this approach, we obtain $\rho_{v}/\rho_{g}$ of 1.17 for the study area. The main reasons for the low accuracy can be attributed to two factors. Firstly, similar to using experienced value, calculating a single value for the entire Harvard Forest will cause abnormal results due to the complexity of the forest canopy. Secondly, the quality of the LiDAR can greatly affect the results. Such as areas in low point density in canopy that do not reflect enough energy (Chauve et al., 2009). Combining the spaceborne spectral data and airborne LiDAR data to calculate the reflectance ratio can use high-quality spectral data to a certain extent to eliminate the abnormal phenomenon of reflectance ratio caused by the abnormal collection of some LiDAR footprints. Compared with the above two methods, the gridded $\rho_{v}/\rho_{g}$ calculation method we proposed considers the influence of these factors. By combining spaceborne spectral data and airborne LiDAR waveform to calculate the $\rho_{v}/\rho_{g}$ , the influence of abnormal waveforms on the value of $\rho_{v}/\rho_{g}\;$ can be eliminated to some extent. At the same time, laborious ground truth measurements of reflectance are no longer needed (Yang et al., 2006). Furthermore, dividing the study area into grids and calculating the average $\rho_{v}/\rho_{g}$ in each grid can not only further eliminate abnormal values through statistical method, but also calculate different $\rho_{v}/\rho_{g}$ in view of the canopy heterogeneity in large-scale complex forest. It can be found from the LAI maps of the three methods that the method of taking a constant value would lead to higher LAI values in the areas of dense vegetation, which is due to the fact that the actual $\rho_{v}/\rho_{g}\;$ in these areas are higher than the determined value of the model. LAI results obtained by the method we proposed are basically within 7, with only a few outliers. The results also confirm the validity of the proposed method.

In addition to the inversion based on the GORT physical model, the LAI inversion based on the empirical NDVI-LAI relationship (Turner et al., 1999) is also carried out based on the spectral data. The results show that the inversion accuracy of the empirical model using spectral data (correlation of 0.48 and R^2^ of 0.25) is much lower than that of the physical model using LiDAR waveforms. The empirical model needs a certain amount of truth values to ensure the accuracy of the inversion equation. However, there are only 48 small plots in the study area, which in theory can cause large errors when used for empirical model construction and verification. Also, the existing plots of Harvard Forest HEM and LPH Towers are concentrated in a small area, which cannot well represent the NDVI-LAI relationship of the entire study area. It is the main reason for the lower precision., Our results show that the strategy we proposed in this study is viable for predicting forest LAI. The combined multispectral imagery and LiDAR waveform can improve the input parameter $\rho_{v}/\rho_{g}$ of the GORT model and contribute to prediction accuracy of LAI.

### 4.4 Limitations

While this study has successfully achieved the joint inversion of spectral and LiDAR data for LAI estimation based on a physical model, there are still some limitations. Firstly, due to the limited number of ground measurement points in our study area, we are unable to achieve the fusion of LiDAR and spectral data for LAI retrieval based on empirical models, and compare it with the results from physical models. Nevertheless, this also partly demonstrates the advantages of developing data fusion inversion based on physical models, which helps us reduce reliance on ground measurements, lower manual labor costs, and facilitates the widespread application of large-scale regions. Secondly, while both LiDAR data and spectral data are commonly employed for retrieving canopy cover, there remains a disparity between the values obtained through these two methods (Smith et al., 2009; Li et al., 2023). This discrepancy, though overlooked in this experiment, may cause errors in inversion. It might be one of the contributing factors to the limited precision in LAI inversion. Research is warranted in future experiments to address this issue and enhance the inversion accuracy. Additionally, due to time and resource constraints, we do not validate the effectiveness of this method in regional scale. In future research, we will further explore the contribution of data fusion to LAI based on theoretical analysis.

## 5 Conclusion

Spectral imagery and full-waveform LiDAR data can provide reflectance information and echo energy information reflecting the vertical structure of the forest canopy respectively. Joint active and passive remote sensing data has great potential for accurate inversion of forest canopy LAI. Our research is one of the few attempts to derive LAI using both spectral imagery and LiDAR waveform based on physical model retrieval rather than through empirical methods. We proposed a useful data-joint LAI inversion strategy based on the GORT model using LiDAR waveform and spectral data. For the large-scale heterogeneous forest, we further accurately extracted the waveform energy parameters as the model input data and optimized the model input parameter canopy/ground reflectivity ratio to improve the inversion accuracy. The results show that comparing with only using LiDAR or spectral imagery, the LAI calculated by the proposed strategy using both LiDAR waveform and spectral imagery has a higher accuracy, indicating the effectiveness of the proposed strategy. Overall, our study confirms that optimizing the input parameter and data of the model for the study area can help improve the inversion accuracy, and the combined LiDAR waveform and multispectral imagery have potential for improving prediction accuracies of LAI.

## Data availability statement

The original contributions presented in the study are included in the article/supplementary material. Further inquiries can be directed to the corresponding author.

## Author contributions

Conceptualization, ZS and SS. Methodology, ZS and SS. Formal analysis, ZS and LX. Investigation, ZS and BW. Data curation, BC and QX. Writing—original draft preparation, ZS. Writing—review and editing, JS and SS. Supervision, WG. Project administration, WG. Funding acquisition, WG and SS. All authors contributed to the article and approved the submitted version.

## References

[B1] Armston J. Disney M. Lewis P. Scarth P. Phinn S. Lucas R.. (2013). Direct retrieval of canopy gap probability using airborne waveform lidar. Remote Sens. Environ. 134, 24–38. doi: 10.1016/j.rse.2013.02.021

[B2] Barclay H. J. Goodman D. (2000). Conversion of total to projected leaf area index in conifers. Can. J. Botany-Revue Can. Botanique 78, 447–454. doi: 10.1139/cjb-78-4-447

[B3] Blair J. B. Hofton. M. (2020). LVIS Classic L1B Geolocated Return Energy Waveforms, Version 1 (Boulder, Colorado USA: NASA National Snow and Ice Data Center Distributed Active Archive Center). doi: 10.5067/O8UCOA2D6ZE3

[B4] Blair J. B. Rabine D. L. Hofton M. A. (1999). The Laser Vegetation Imaging Sensor: a medium-altitude, digitisation-only, airborne laser altimeter for mapping vegetation and topography. Isprs J. Photogrammetry Remote Sens. 54, 115–122. doi: 10.1016/S0924-2716(99)00002-7

[B5] Chauve A. Vega C. Durrieu S. Bretar F. Allouis T. Deseilligny M. P.. (2009). Advanced full-waveform lidar data echo detection: Assessing quality of derived terrain and tree height models in an alpine coniferous forest. Int. J. Remote Sens. 30, 5211–5228. doi: 10.1080/01431160903023009

[B6] Chen J. M. (1996). Optically-based methods for measuring seasonal variation of leaf area index in boreal conifer stands. Agric. For. Meteorol. 80, 135–163. doi: 10.1016/0168-1923(95)02291-0

[B7] Chen J. M. Black T. A. (1992). Defining leaf-area index for non-flat leaves. Plant Cell Environ. 15, 421–429. doi: 10.1111/j.1365-3040.1992.tb00992.x

[B8] Clevers J. vanLeeuwen H. J. C. (1996). Combined use of optical and microwave remote sensing data for crop growth monitoring. Remote Sens. Environ. 56, 42–51. doi: 10.1016/0034-4257(95)00227-8

[B9] Drusch M. Del Bello U. Carlier S. Colin O. Fernandez V. Gascon F.. (2012). Sentinel-2: ESA's optical high-resolution mission for GMES operational services. Remote Sens. Environ. 120, 25–36. doi: 10.1016/j.rse.2011.11.026

[B10] Fang H. L. Baret F. Plummer S. Schaepman-Strub G. (2019). An overview of global leaf area index (LAI): methods, products, validation, and applications. Rev. Geophys. 57, 739–799. doi: 10.1029/2018RG000608

[B11] Fiala A. C. S. Garman S. L. Gray A. N. (2006). Comparison of five canopy cover estimation techniques in the western Oregon Cascades. For. Ecol. Manage. 232, 188–197. doi: 10.1016/j.foreco.2006.05.069

[B12] Fieber K. D. Davenport I. J. Tanase M. A. Ferryman J. M. Gurney R. J. Walker J. P.. (2014). Effective LAI and CHP of a single tree from small-footprint full-waveform liDAR. IEEE Geosci. Remote Sens. Lett. 11, 1634–1638. doi: 10.1109/LGRS.2014.2303500

[B13] Gastellu-Etchegorry J. P. Lauret N. Yin T. G. Landier L. Kallel A. Malenovsky Z.. (2017). DART: recent advances in remote sensing data modeling with atmosphere, polarization, and chlorophyll fluorescence. IEEE J. Selected Topics Appl. Earth Observations Remote Sens. 10, 2640–2649. doi: 10.1109/JSTARS.2017.2685528

[B14] Gastellu-Etchegorry J. P. Yin T. G. Lauret N. Grau E. Rubio J. Cook B. D.. (2016). Simulation of satellite, airborne and terrestrial LiDAR with DART (I): Waveform simulation with quasi-Monte Carlo ray tracing. Remote Sens. Environ. 184, 418–435. doi: 10.1016/j.rse.2016.07.010

[B15] Houborg R. Soegaard H. Boegh E. (2007). Combining vegetation index and model inversion methods for the extraction of key vegetation biophysical parameters using Terra and Aqua MODIS reflectance data. Remote Sens. Environ. 106, 39–58. doi: 10.1016/j.rse.2006.07.016

[B16] Hu Q. Yang J. Xu B. Huang J. Memon M. S. Yin G.. (2020). Evaluation of global decametric-resolution LAI, FAPAR and FVC estimates derived from sentinel-2 imagery. Remote Sens. 12, 912. doi: 10.3390/rs12060912

[B17] Jiang H. L. Cheng S. Y. Yan G. J. Kuusk A. Hu R. H. Tong Y. Y.. (2022). Clumping effects in leaf area index retrieval from large-footprint full-waveform liDAR. IEEE Trans. Geosci. Remote Sens. 60, 1–20. doi: 10.1109/TGRS.2021.3118925

[B18] Kennedy B. E. King D. J. Duffe J. (2020). Comparison of empirical and physical modelling for estimation of biochemical and biophysical vegetation properties: field scale analysis across an arctic bioclimatic gradient. Remote Sens. 12 (18), 3073. doi: 10.3390/rs12183073

[B19] Korhonen L. Hadi Packalen P. Rautiainen M. (2017). Comparison of Sentinel-2 and Landsat 8 in the estimation of boreal forest canopy cover and leaf area index. Remote Sens. Environ. 195, 259–274. doi: 10.1016/j.rse.2017.03.021

[B20] Korhonen L. Korpela I. Heiskanen J. Maltamo M. (2011). Airborne discrete-return LIDAR data in the estimation of vertical canopy cover, angular canopy closure and leaf area index. Remote Sens. Environ. 115, 1065–1080. doi: 10.1016/j.rse.2010.12.011

[B21] Lefsky M. A. Harding D. Cohen W. B. Parker G. Shugart H. H. (1999). Surface lidar remote sensing of basal area and biomass in deciduous forests of eastern Maryland, USA. Remote Sens. Environ. 67, 83–98. doi: 10.1016/S0034-4257(98)00071-6

[B22] Li L. Y. Mu X. H. Jiang H. L. Chianucci F. Hu R. H. Song W. J.. (2023). Review of ground and aerial methods for vegetation cover fraction (fCover) and related quantities estimation: definitions, advances, challenges, and future perspectives. Isprs J. Photogrammetry Remote Sens. 199, 133–156. doi: 10.1016/j.isprsjprs.2023.03.020

[B23] Li X. W. Strahler A. H. Woodcock C. E. (1995). A hybrid geometric optical-radiative transfer approach for modeling albedo and directional reflectance of discontinuous canopies. IEEE Trans. Geosci. Remote Sens. 33, 466–480. doi: 10.1109/TGRS.1995.8746028

[B24] Liang L. Di L. P. Zhang L. P. Deng M. X. Qin Z. H. Zhao S. H.. (2015). Estimation of crop LAI using hyperspectral vegetation indices and a hybrid inversion method. Remote Sens. Environ. 165, 123–134. doi: 10.1016/j.rse.2015.04.032

[B25] Liu K. Zhou Q. B. Wu W. B. Xia T. Tang H. J. (2016). Estimating the crop leaf area index using hyperspectral remote sensing. J. Integr. Agric. 15, 475–491. doi: 10.1016/S2095-3119(15)61073-5

[B26] Luo S. Z. Wang C. Li G. C. Xi X. H. (2013). Retrieving leaf area index using ICESat/GLAS full-waveform data. Remote Sens. Lett. 4, 745–753. doi: 10.1080/2150704X.2013.790573

[B27] Luo S. Z. Wang C. Xi X. H. Nie S. Fan X. Y. Chen H. Y.. (2019). Combining hyperspectral imagery and LiDAR pseudo-waveform for predicting crop LAI, canopy height and above-ground biomass. Ecol. Indic. 102, 801–812. doi: 10.1016/j.ecolind.2019.03.011

[B28] Ma H. Song J. L. Wang J. D. (2015). Forest canopy LAI and vertical FAVD profile inversion from airborne full-waveform liDAR data based on a radiative transfer model. Remote Sens. 7, 1897–1914. doi: 10.3390/rs70201897

[B29] Ma H. Song J. L. Wang J. D. Xiao Z. Q. Fu Z. (2014). Improvement of spatially continuous forest LAI retrieval by integration of discrete airborne LiDAR and remote sensing multi-angle optical data. Agric. For. Meteorol. 189, 60–70. doi: 10.1016/j.agrformet.2014.01.009

[B30] Ma Q. Su Y. J. Guo Q. H. (2017). Comparison of canopy cover estimations from airborne liDAR, aerial imagery, and satellite imagery. IEEE J. Selected Topics Appl. Earth Observations Remote Sens. 10, 4225–4236. doi: 10.1109/JSTARS.2017.2711482

[B31] Mallet C. Bretar F. (2009). Full-waveform topographic lidar: State-of-the-art. Isprs J. Photogrammetry Remote Sens. 64, 1–16. doi: 10.1016/j.isprsjprs.2008.09.007

[B32] Mananze S. Pocas I. Cunha M. (2018). Retrieval of maize leaf area index using hyperspectral and multispectral data. Remote Sens. 10 (12), 1942. doi: 10.3390/rs10121942

[B33] Moran C. J. Kane V. R. Seielstad C. A. (2020). Mapping forest canopy fuels in the western United States with liDAR–landsat covariance. Remote Sens. 12 (6), 1000. doi: 10.3390/rs12061000

[B34] Morsdorf F. Kotz B. Meier E. Itten K. I. Allgower B. (2006). Estimation of LAI and fractional cover from small footprint airborne laser scanning data based on gap fraction. Remote Sens. Environ. 104, 50–61. doi: 10.1016/j.rse.2006.04.019

[B35] Myneni R. B. Nemani R. R. Running S. W. (1997). Estimation of global leaf area index and absorbed par using radiative transfer models. IEEE Trans. Geosci. Remote Sens. 35, 1380–1393. doi: 10.1109/36.649788

[B36] Neinavaz E. Skidmore A. K. Darvishzadeh R. Groen T. A. (2016). Retrieval of leaf area index in different plant species using thermal hyperspectral data. Isprs J. Photogrammetry Remote Sens. 119, 390–401. doi: 10.1016/j.isprsjprs.2016.07.001

[B37] Ni W. G. Li X. W. Woodcock C. E. Caetano M. R. Strahler A. H. (1999). An analytical hybrid GORT model for bidirectional reflectance over discontinuous plant canopies. IEEE Trans. Geosci. Remote Sens. 37, 987–999. doi: 10.1109/36.752217

[B38] Ni-Meister W. Jupp D. L. B. Dubayah R. (2001). Modeling lidar waveforms in heterogeneous and discrete canopies. IEEE Trans. Geosci. Remote Sens. 39, 1943–1958. doi: 10.1109/36.951085

[B39] Ni-Meister W. Lee S. Y. Strahler A. H. Woodcock C. E. Schaaf C. Yao T. A.. (2010). Assessing general relationships between aboveground biomass and vegetation structure parameters for improved carbon estimate from lidar remote sensing. J. Geophys. Research-Biogeosciences 115. doi: 10.1029/2009JG000936

[B40] Orwig D. Hadley J. (2022). “Leaf Area Index at Harvard Forest HEM and LPH Towers since 1998 ver 23. EDI (Environmental Data Initiative) Data Portal. doi: 10.6073/pasta/912d4da0d326da63d82e93de68ca5ad4

[B41] Pan M. Wood E. F. Wojcik R. Mccabe M. F. (2008). Estimation of regional terrestrial water cycle using multi-sensor remote sensing observations and data assimilation. Remote Sens. Environ. 112, 1282–1294. doi: 10.1016/j.rse.2007.02.039

[B42] Persson Å. Söderman U. Töpel J. Ahlberg S. (2005). Visualization and analysis of full-waveform airborne laser scanner data. Int. Arch. Photogrammetry Remote Sens. Spatial Inf. Sci. 36, 103–108.

[B43] Pope G. Treitz P. (2013). Leaf area index (LAI) estimation in boreal mixedwood forest of ontario, Canada using light detection and ranging (LiDAR) and worldView-2 imagery. Remote Sens. 5, 5040–5063. doi: 10.3390/rs5105040

[B44] Potithep S. Nagai S. Nasahara K. N. Muraoka H. Suzuki R. (2013). Two separate periods of the LAI-VIs relationships using in *situ* measurements in a deciduous broadleaf forest. Agric. For. Meteorol. 169, 148–155. doi: 10.1016/j.agrformet.2012.09.003

[B45] Qu Y. H. Han W. C. Ma M. G. (2015). Retrieval of a temporal high-resolution leaf area index (LAI) by combining MODIS LAI and ASTER reflectance data. Remote Sens. 7, 195–210. doi: 10.3390/rs70100195

[B46] Silvan-Cardenas J. L. Wang L. (2010). Retrieval of subpixel Tamarix canopy cover from Landsat data along the Forgotten River using linear and nonlinear spectral mixture models. Remote Sens. Environ. 114, 1777–1790. doi: 10.1016/j.rse.2010.04.003

[B47] Smith A. M. S. Falkowski M. J. Hudak A. T. Evans J. S. Robinson A. P. Steele C. M. (2009). A cross-comparison of field, spectral, and lidar estimates of forest canopy cover. Can. J. Remote Sens. 35, 447–459. doi: 10.5589/m09-038

[B48] Sun Y. H. Qin Q. M. Ren H. Z. Zhang Y. (2022). Decameter cropland LAI/FPAR estimation from sentinel-2 imagery using google earth engine. IEEE Trans. Geosci. Remote Sens. 60, 4400614. doi: 10.1109/TGRS.2021.3052254

[B49] Tang H. Dubayah R. Brolly M. Ganguly S. Zhang G. (2014). Large-scale retrieval of leaf area index and vertical foliage profile from the spaceborne waveform lidar (GLAS/ICESat). Remote Sens. Environ. 154, 8–18. doi: 10.1016/j.rse.2014.08.007

[B50] Tang H. Dubayah R. Swatantran A. Hofton M. Sheldon S. Clark D. B.. (2012). Retrieval of vertical LAI profiles over tropical rain forests using waveform lidar at La Selva, Costa Rica. Remote Sens. Environ. 124, 242–250. doi: 10.1016/j.rse.2012.05.005

[B51] Thomas V. Noland T. Treitz P. Mccaughey J. H. (2011). Leaf area and clumping indices for a boreal mixed-wood forest: lidar, hyperspectral, and Landsat models. Int. J. Remote Sens. 32, 8271–8297. doi: 10.1080/01431161.2010.533211

[B52] Tseng Y. H. Lin L. P. Wang C. K. (2016). Mapping CHM and LAI for heterogeneous forests using airborne full-waveform liDAR data. Terrestrial Atmospheric Oceanic Sci. 27, 537–548. doi: 10.3319/TAO.2016.01.29.04(ISRS)

[B53] Turner D. P. Cohen W. B. Kennedy R. E. Fassnacht K. S. Briggs J. M. (1999). Relationships between leaf area index and Landsat TM spectral vegetation indices across three temperate zone sites. Remote Sens. Environ. 70, 52–68. doi: 10.1016/S0034-4257(99)00057-7

[B54] Verrelst J. Camps-Valls G. Munoz-Mari J. Rivera J. P. Veroustraete F. Clevers J.. (2015). Optical remote sensing and the retrieval of terrestrial vegetation bio-geophysical properties - A review. Isprs J. Photogrammetry Remote Sens. 108, 273–290. doi: 10.1016/j.isprsjprs.2015.05.005

[B55] Verrelst J. Malenovsky Z. van der Tol C. Camps-Valls G. Gastellu-Etchegorry J. P. Lewis P.. (2019). Quantifying vegetation biophysical variables from imaging spectroscopy data: A review on retrieval methods. Surveys Geophys. 40, 589–629. doi: 10.1007/s10712-018-9478-y PMC7613341 36081834

[B56] Wagner W. Ullrich A. Ducic V. Melzer T. Studnicka N. (2006). Gaussian decomposition and calibration of a novel small-footprint full-waveform digitising airborne laser scanner. Isprs J. Photogrammetry Remote Sens. 60, 100–112. doi: 10.1016/j.isprsjprs.2005.12.001

[B57] Wang Y. Fang H. L. (2020). Estimation of LAI with the liDAR technology: A review. Remote Sens. 12 (20), 3457. doi: 10.3390/rs12203457

[B58] Wang Y. Fang H. L. Zhang Y. H. Li S. J. Pang Y. Ma T.. (2023). Retrieval and validation of vertical LAI profile derived from airborne and spaceborne LiDAR data at a deciduous needleleaf forest site. Giscience Remote Sens. 60 (1), 2214987. doi: 10.1080/15481603.2023.2214987

[B59] Weiss M. Jacob F. Duveiller G. (2020). Remote sensing for agricultural applications: A meta-review. Remote Sens. Environ. 236, 111402. doi: 10.1016/j.rse.2019.111402

[B60] Xu J. Quackenbush L. J. Volk T. A. Im J. (2020). Forest and crop leaf area index estimation using remote sensing: research trends and future directions. Remote Sens. 12 (18), 2934. doi: 10.3390/rs12182934

[B61] Xu L. Shi S. Gong W. Shi Z. X. Qu F. F. Tang X. T.. (2022). Improving leaf chlorophyll content estimation through constrained PROSAIL model from airborne hyperspectral and LiDAR data. Int. J. Appl. Earth Observation Geoinformation 115, 103128. doi: 10.1016/j.jag.2022.103128

[B62] Yang W. Z. Tan B. Huang D. Rautiainen M. Shabanov N. V. Wang Y.. (2006). MODIS leaf area index products: From validation to algorithm improvement. IEEE Trans. Geosci. Remote Sens. 44, 1885–1898. doi: 10.1109/TGRS.2006.871215

[B63] Yang X. B. Wang C. Pan F. F. Nie S. Xi X. H. Luo S. Z. (2019). Retrieving leaf area index in discontinuous forest using ICESat/GLAS full-waveform data based on gap fraction model. Isprs J. Photogrammetry Remote Sens. 148, 54–62. doi: 10.1016/j.isprsjprs.2018.12.010

[B64] Yang G. J. Zhao C. J. Liu Q. Huang W. J. Wang J. H. (2011). Inversion of a radiative transfer model for estimating forest LAI from multisource and multiangular optical remote sensing data. IEEE Trans. Geosci. Remote Sens. 49, 988–1000. doi: 10.1109/TGRS.2010.2071416

[B65] Zhang F. Hassanzadeh A. Kikkert J. Pethybridge S. J. Van Aardt J. (2022). Evaluation of leaf area index (LAI) of broadacre crops using UAS-based liDAR point clouds and multispectral imagery. IEEE J. Selected Topics Appl. Earth Observations Remote Sens. 15, 4027–4044. doi: 10.1109/JSTARS.2022.3172491

[B66] Zhang Z. J. Xie H. Tong X. H. Zhang H. W. Liu Y. Li B. B. (2020). Denoising for satellite laser altimetry full-waveform data based on EMD-Hurst analysis. Int. J. Digital Earth 13, 1212–1229. doi: 10.1080/17538947.2019.1698665

[B67] Zhang Y. Yang Y. Z. Zhang Q. W. Duan R. Q. Liu J. Q. Qin Y. C.. (2023). Toward multi-stage phenotyping of soybean with multimodal UAV sensor data: A comparison of machine learning approaches for leaf area index estimation. Remote Sens. 15 (1), 7. doi: 10.3390/rs15010007

[B68] Zhao F. Yang X. Y. Schull M. A. Roman-Colon M. O. Yao T. Wang Z. S.. (2011). Measuring effective leaf area index, foliage profile, and stand height in New England forest stands using a full-waveform ground-based lidar. Remote Sens. Environ. 115, 2954–2964. doi: 10.1016/j.rse.2010.08.030

[B69] Zheng G. Moskal L. M. (2009). Retrieving leaf area index (LAI) using remote sensing: theories, methods and sensors. Sensors 9, 2719–2745. doi: 10.3390/s90402719 22574042 PMC3348792

[B70] Zhou G. Q. Deng R. H. Zhou X. Long S. H. Li W. H. Lin G. C.. (2022). Gaussian inflection point selection for liDAR hidden echo signal decomposition. IEEE Geosci. Remote Sens. Lett. 19, 1–5. doi: 10.1109/LGRS.2021.3107438

[B71] Zhou G. Q. Long S. H. Xu J. S. Zhou X. Song B. Deng R. H.. (2021). Comparison analysis of five waveform decomposition algorithms for the airborne liDAR echo signal. IEEE J. Selected Topics Appl. Earth Observations Remote Sens. 14, 7869–7880. doi: 10.1109/JSTARS.2021.3096197

[B72] Zhou X. J. Wang P. X. Tansey K. Zhang S. Y. Li H. M. Tian H. R. (2020). Reconstruction of time series leaf area index for improving wheat yield estimates at field scales by fusion of Sentinel-2,-3 and MODIS imagery. Comput. Electron. Agric. 177, 105692. doi: 10.1016/j.compag.2020.105692

